# The Fractalkine Receptor CX_3_CR1 Links Lymphocyte Kinetics in CMV-Seropositive Patients and Acute Myocardial Infarction With Adverse Left Ventricular Remodeling

**DOI:** 10.3389/fimmu.2021.605857

**Published:** 2021-05-05

**Authors:** Luke Spray, Catherine Park, Suzanne Cormack, Ashfaq Mohammed, Pedram Panahi, Stephen Boag, Karim Bennaceur, Kateryna Sopova, Gavin Richardson, Verena M. Stangl, Lavinia Rech, Peter P. Rainer, Gustavo Campos Ramos, Ulrich Hofmann, Konstantinos Stellos, Ioakim Spyridopoulos

**Affiliations:** ^1^ Cardiology Department, Freeman Hospital, Newcastle upon Tyne, United Kingdom; ^2^ Translational and Clinical Research Institute, Cardiovascular Biology and Medicine, Newcastle University, Newcastle-upon-Tyne, United Kingdom; ^3^ Biosciences Institute, Cardiovascular Biology and Medicine, Newcastle University, Newcastle-upon-Tyne, United Kingdom; ^4^ Diagnostic and Research Institute of Pathology, Medical University of Graz, Graz, Austria; ^5^ Division of Cardiology, Medical University of Graz, Graz, Austria; ^6^ BioTechMed Graz, Graz, Austria; ^7^ Department of Internal Medicine I and Comprehensive Heart Failure Center, University Hospital Würzburg, Würzburg, Germany

**Keywords:** acute myocardial infarction, CX3CR1, cytomegalovirus, cardiac MRI, remodeling, T-lymphocytes

## Abstract

**Aims:**

Latent cytomegalovirus (CMV) infection is associated with adverse cardiovascular outcomes. Virus-specific CX_3_CR1^+^ effector memory T-cells may be instrumental in this process due to their pro-inflammatory properties. We investigated the role of CX_3_CR1 (fractalkine receptor) in CMV-related lymphocyte kinetics and cardiac remodeling in patients with ST-elevation myocardial infarction (STEMI) undergoing primary percutaneous coronary intervention (pPCI).

**Methods and Results:**

We retrospectively analysed lymphocyte count, troponin, and survival in 4874 STEMI/pPCI patients, evaluated lymphocyte kinetics during reperfusion in a prospective cohort, and obtained sequential cardiac MRI (cMRI) to assess remodeling. Pre-reperfusion lymphopenia independently predicted mortality at 7.5 years. Prior to reperfusion, CCR7^+^ T-lymphocytes appeared to be depleted. After reperfusion, T-lymphocytes expressing CX_3_CR1 were depleted predominantly in CMV-seropositive patients. During ischaemia/reperfusion, a drop in CX_3_CR1^+^ T-lymphocytes was significantly linked with microvascular obstruction in CMV+ patients, suggesting increased fractalkine-receptor interaction. At 12 weeks, CMV+ patients displayed adverse LV remodeling.

**Conclusion:**

We show that lymphopenia occurs before and after reperfusion in STEMI by different mechanisms and predicts long-term outcome. In CMV+ patients, increased fractalkine induction and sequestration of CX_3_CR1^+^ T-cells may contribute to adverse remodeling, suggesting a pro-inflammatory pathomechanism which presents a novel therapeutic target.

## Introduction

The immunological component of atherosclerosis and coronary artery disease has garnered increasing interest in the last decade. Recent phase III trials of anti-inflammatory agents in humans show promising, although inconsistent results (1–3). The CANTOS trial demonstrated the potential for targeting specific pathways in the innate immune system (1), but similarly targeted inhibition of the adaptive immune system has not yet yielded such favourable results (4).

Our group has shown that T-lymphocytes contribute to myocardial ischaemia/reperfusion injury after primary percutaneous coronary intervention (pPCI) for ST-elevation myocardial infarction (STEMI), with the fractalkine receptor, CX_3_CR1, playing a central role (5). We have also shown that latent cytomegalovirus (CMV) infection confers worse cardiovascular mortality in elderly patients (6). CMV, a ubiquitous herpes virus, inflates the T-lymphocyte compartment with CMV-specific, cytotoxic T-cells (5, 7–9), which have been shown to induce endothelial damage through induction of fractalkine (the CX_3_CR1 ligand) (9, 10).

CX_3_CR1 is a surface marker for monocytes and effector lymphocytes, specifically natural killer (NK) cells and cytotoxic T-lymphocytes, and can induce the release of inflammatory chemokines (10–13).

Having previously shown that T-lymphocytes are crucial mediators of the response to myocardial ischaemia and reperfusion (5), we are now the first to analyse how this response differs in the CMV-acquainted lymphocyte compartment, and how it affects remodeling after myocardial infarction.

## Methods

Details of the major resources and detailed methods can be found in the online-only Data Supplement.

### Patient Populations

**Figure 1:**
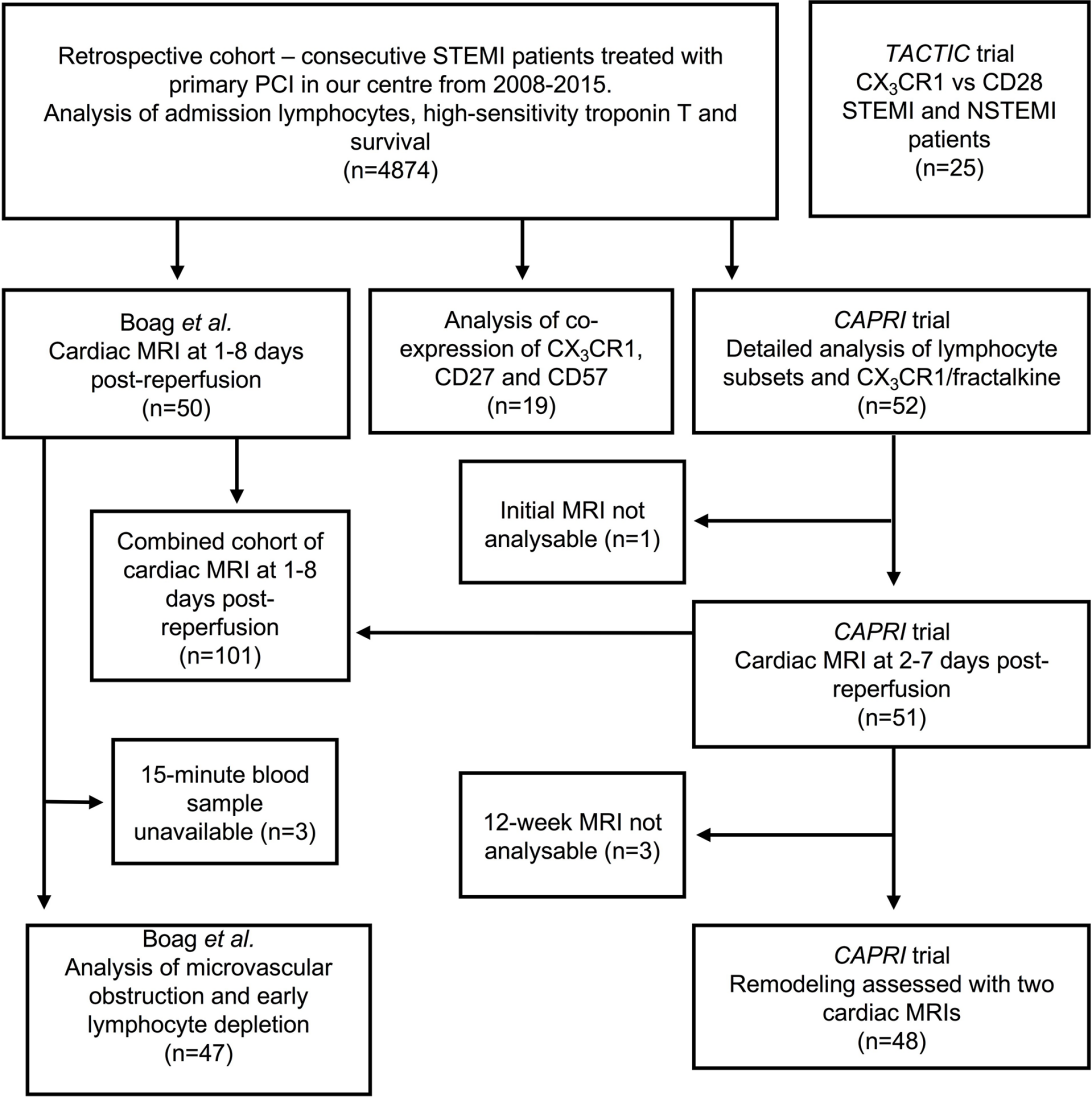
Overview of patients with acute MI used in this study. Three of the four prospective cohorts were recruited between 2008 and 2015, so these patients are also included in the retrospective analysis. The TACTIC trial recruited patients after 2015, so these are not included in the retrospective cohort. cMRIs were unanalysable either because of technical problems with the scanner or poor image quality (e.g. due to atrial fibrillation). Acute cMRIs from Boag et al. and CAPRI were analysed together. We also describe data from 10 healthy control patients, and 21 explanted human hearts, not shown in this diagram.

This study utilises several different cohorts of patients. We retrospectively analysed 4874 consecutive STEMI/pPCI patients from our centre between 2008 and 2015. We analysed lymphocytes and CX_3_CR1 in whole blood throughout reperfusion in 52 STEMI/pPCI patients recruited for *CAPRI* (Ciclosporin to Reduce Reperfusion Injury in Primary PCI Clinical Trial) (14) (demographics in **Table 1**), and analysed three further prospective cohorts of STEMI/pPCI patients, and 10 healthy controls. These populations are shown schematically in **Figure 1**.

**Table 1:** Demographic characteristics of all patients with a cMRI both at 2-7 days and at 12 weeks, split into CMV positive and negative (n=48).

	CMV Positive (n=29)	CMV Negative (n=19)	p value
*Age (years)*	67.0 ± 1.9	61.6 ± 2.3	0.08
*Male*	23 (79.3)	17 (89.5)	0.45
*BMI (kg/m^2^)*	28.9 ± 0.8	26.4 ± 1.8	0.16
*Past CAD*	2 (6.9)	1 (5.3)	>0.99
*Family history of CAD*	11 (37.9)	7 (36.8)	>0.99
*Hypertension*	5 (17.2)	3 (15.8)	>0.99
*Hypercholesterolaemia*	3 (10.3)	0 (0)	0.27
*Diabetes mellitus*	2 (6.9)	2 (10.5)	>0.99
*Current smoker*	4 (13.8)	7 (36.8)	0.09
*Anterior MI*	7 (24.1)	6 (31.6)	0.74
*Peak troponin (ng/L)*	3804 ± 528	4716 ± 747	0.37
*Acute infarct size (% of LV)*	10.2 ± 1.4	13.3. ± 3.0	0.60
*Pre-admission medication*			
*Aspirin*	3 (10.3)	1 (5.3)	>0.99
*β-blocker*	1 (3.4)	1 (5.3)	>0.99
*ACE-inhibitor/ARB*	4 (13.8)	2 (10.5)	>0.99
*Diuretic*	3 (10.3)	1 (5.3)	>0.99
*Statin*	8 (27.6)	2 (10.5)	0.28
*Calcium channel blocker*	3 (10.3)	2 (10.5)	>0.99
*Procedure details*			
*Onset to balloon time (min)*	191 ± 16.7	200 ± 23.3	0.95
*>1 vessel treated*	3 (10.3)	3 (15.8)	0.67
*Flow pre-PCI (TIMI 0/1/2/3)*	24/5/0/0	17/2/0/0	0.69
*Flow post-PCI (TIMI 0/1/2/3)*	0/2/0/27	0/0/0/19	0.51

Four further patients were used in detailed lymphocyte analysis, and their inclusion does not meaningfully alter the demographics presented. Continuous variables are presented as mean ± SEM. Discrete variables are presented as count (percentage). p values were determined with the Mann-Whitney U test for continuous variables and Fischer’s exact test for discrete. P values <0.05 were considered statistically significant. BMI, Body Mass Index; CAD, coronary artery disease; ACE, angiotensin-converting enzyme; ARB, angiotensin receptor blocker; TIMI, thrombolysis in myocardial infarction.

### TruCount and Multicolour Flow Cytometry

Different multicolour assays were used as previously described (5). A 6-colour assay measured the expression of CD3, CD4, CD8, CD45RA, CCR7 and CX_3_CR1 in the sample (**Supplemental Figure 1A**). We gated T-lymphocytes (CD3^+^) first into CD4^+^ and CD8^+^ T cells, then into four subsets of each by expression of CCR7, the chemokine receptor for CCL19/21, and CD45RA (**Supplemental Figure 1A**), as previously described by Sallusto (15). We quantified CX_3_CR1 mean fluorescence intensity (MFI) in all subsets. Using 8-colour assays, we measured co-expression of CD27 and CD57 MFI (n=19), and CD28 MFI (n=25) (**Supplemental Figures 1B, C** and **4B**).

### CMV Serostatus

CMV serostatus in all cohorts was determined with Roche Elecsys CMV IgG immunoassay. CMV seropositivity was defined as an IgG index >1.0 U/mL, as per manufacturer’s instructions.

### Cardiac Magnetic Resonance Imaging

In *CAPRI*, acute cMRI was obtained at 2-7 days post-reperfusion in 51 patients, and repeat cMRI at 12 weeks was obtained in 48 patients to assess remodeling. In another study 50 cMRI scans were acquired 1-8 days post-reperfusion (5), providing a total of 101 acute cMRIs. Parameters of interest were infarct size as a percentage of the left ventricle (LV), LV ejection fraction, end-diastolic LV volume, end-systolic LV volume and microvascular obstruction (MVO), and were measured as previously described and validated (5).

### Immunohistochemistry of Human Hearts

Left ventricular myocardial tissue samples were obtained from failing and non-failing human heart explants (n=21), the latter that were not accepted for transplantation. Eight were female, mean age was 62 ± 12 years, mean left ventricular ejection fraction 64 ± 7%. Tissue samples were collected at the time of explantation, formalin fixed, and paraffin embedded. Immunohistochemistry was performed using anti-human CD3 primary antibody (Dako, G A503) according to manufacturer’s recommendations. Quantification was performed manually by a pathologist blinded to CMV serostatus.

### Study Approval

Favourable ethical opinions were received for the *CAPRI* study (National Research Ethics Committee North-East – Newcastle and North Tyneside 2; REC reference: 14/NE/1070), the cohort recruited by Boag *et al.* (National Research Ethics Service Committee North East; REC reference: 12/NE/0322), the cohort of 19 STEMI patients (National Research Ethics Service Committee East Midlands – Derby; REC reference: 15/EM/0072) and the *TACTIC* study (National Research Ethics Committee North-East – Newcastle and North Tyneside 1 Research Ethics Committee; REC reference: 18/NE/0178). *CAPRI* received a clinical trial authorisation from the Medicines and Healthcare products Regulatory Agency. All STEMI/pPCI patients analysed retrospectively had provided written informed consent to their clinical data being included in a database of pPCI procedures to be used for research. The use of human biomaterials in our study of 21 explanted human hearts was approved by the Ethics committee of the Medical University of Graz (20-277 ex 08/09, 26-282 ex 13/14 and 28-508 ex 15/16) and conformed to all pertaining regulations. All studies used complied with the principles laid out in the declaration of Helsinki, and written informed consent was obtained from all participants.

### Statistics

Statistical analysis was performed using SPSS version 26. Graphics were produced using GraphPad Prism version 8. Statistical tests used are given in the figure legends. Data is presented as n (%) for discrete variables, and mean ± SEM for continuous variables, unless otherwise stated. Patients in the retrospective analysis were assigned to quartiles according to admission lymphocytes. Data normality was assessed using the Shapiro-Wilk test. Comparisons between lymphocyte quartile groups were performed with the Kruskal-Wallis test with Dunn’s correction for multiple comparisons for continuous variables or with the Chi-square test for categorical variables. Clinical and laboratory variables that were statistically (p < 0.05) associated with overall mortality by univariate analysis were selected for backwards conditional multivariate Cox-regression analysis (**Supplemental Table 4**). Only variables that were independently associated with mortality by multivariate Cox-regression were selected to compose the final prediction model. Univariate associations of lymphocytes with overall mortality were assessed by Kaplan Meier survival analysis and unadjusted Cox-regression. In the prospective cohort, although no baseline characteristics were significantly associated with CMV serostatus, those which appeared clinically relevant and differed between CMV serogroups were selected for multivariate linear regression analysis of change in end-diastolic volume (**Table 2**) and end-systolic volume (**Supplemental Table 7**).

**Table 2:** Full list of variables entered into multivariate linear regression model for change in end-diastolic volume over 12 weeks.

	Coefficient	95% Confidence interval for coefficient	p value
*Age (years)*	-0.152	-0.964 – 0.659	0.706
*Current smoker*	1.603	-9.228 – 12.433	0.766
*History of hypercholesterolemia*	-8.406	-22.800 – 5.988	0.245
*Statins as premedication*	-6.504	-25.847 – 12.839	0.501
*Peak troponin (ng/mL)*	0.00007	-0.002 – 0.002	0.951
*CMV IgG serostatus*	22.448	7.028 – 37.868	0.005
*Anterior infarct*	14.115	-1.849 – 30.078	0.082

Adjusted coefficients with 95% confidence intervals are shown for all covariates, along with p values for coefficient. n=52 with no missing values.

## Results

### CMV Seropositive Patients Show Adverse Left-Ventricular Remodeling After STEMI

**Figure 2:**
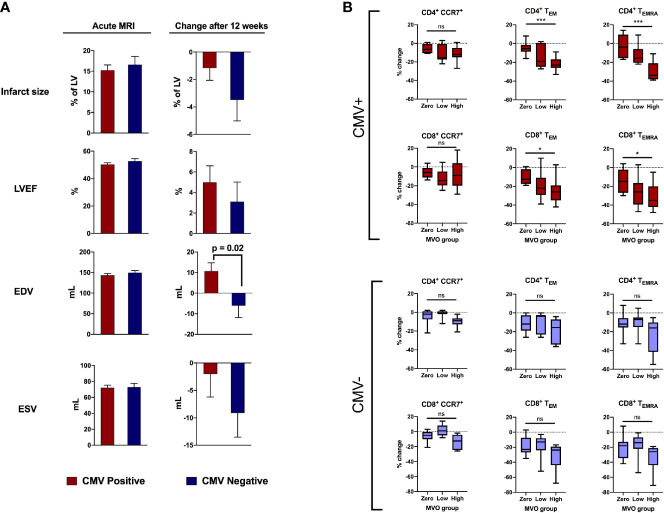
CMV serostatus is associated with adverse remodeling 12 weeks after reperfusion. **(A)** In the acute phase (1-8 days post-reperfusion, n=101), CMV serostatus has no effect on infarct size, LV ejection fraction, end-diastolic volume or end-systolic volume. At 12-week follow-up (n=48), CMV seropositive patients displayed significantly more deterioration in end-diastolic volume (+10.7mL vs -6.1mL, p=0.02). P values determined using the unpaired t-test. **(B)** Relationship between amount of MVO (zero, low or high) and change in T cell subsets between 15-30 minutes post-reperfusion, separately for CMV seropositive and seronegative patients. Box plots display median (central line), 25^th^ and 75^th^ centiles (limits of box), and range (error bars). Statistics refer to differences between MVO groups as indicated (Kruskal-Wallis test with Dunn’s multiple comparisons test). Total n=47; CMV positive n=25 [8 zero MVO, 6 low, 11 high], CMV negative n=22 [9 zero MVO, 7 low, 6 high]). * p<0.05, ***p<0.001; ns, not significant.

We first investigated whether CMV serostatus affected remodeling of the left ventricle in the first 12 weeks after acute STEMI/pPCI (**Figure 2A**). By comparing 101 cMRIs within 8 days of STEMI/pPCI (58 CMV+, 43 CMV-; demographics in **Supplemental Table 5**), we found that CMV serostatus did not affect early infarct size (15.2% of LV in CMV+ patients *vs* 16.6%; p=0.97), LVEF (50.3% *vs* 52.7%; p=0.2), end-systolic volume (72.1ml *vs* 73.0ml; p=0.9), or end-diastolic volume (143.2ml *vs* 149.3ml; p=0.4). Repeat cMRI at 12 weeks (n=48; 29 CMV+, 19 CMV-) allowed us to assess left ventricular remodeling. CMV seropositive patients displayed a significant deterioration in end-diastolic volume (+10.7ml *vs* -6.1ml; p=0.02), but no significant difference in change in end-systolic volume (-2.0ml *vs* -9.1ml; p=0.27). The effect of CMV serostatus on LV remodeling increased after multivariate analysis adjusting for age, smoking status, statin usage, history of hypercholesterolaemia, peak high-sensitivity troponin T and infarct location (anterior vs non-anterior). The adjusted change in EDV in CMV seropositive patients compared to seronegative patients was +22.5mL (95% CI 7.0 – 37.0mL; p=0.005; **Table 2**), and for ESV was +13.6mL (95% CI -1.0 – 28.1; p=0.068; **Supplemental Table 7**). We hypothesised that CMV-related differences in T-lymphocytes drive inflammation after STEMI, leading to adverse remodeling at 12 weeks.

### Early Post-Reperfusion Loss of CCR7-Negative T-Lymphocytes Is Associated With Microvascular Obstruction in CMV Seropositive Patients

**Figure 3:**
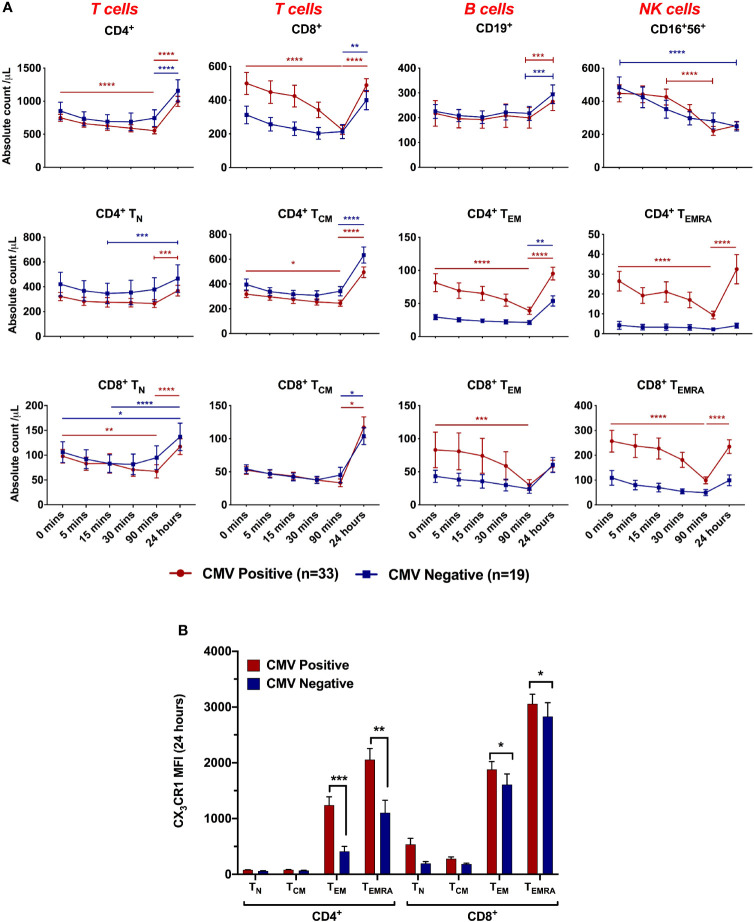
Lymphocyte dynamics during ischaemia/reperfusion depending on CMV serostatus. **(A)** Absolute count of lymphocyte subsets by time point. 0 minutes represents pre-reperfusion, other time points are post-reperfusion. CD4^+^, CD8^+^ and CD16^+^56^+^ cells dropped between 0 and 90 minutes post-reperfusion, and CD4^+^ and CD8^+^ cells rebounded at 24 hours. CMV seropositive patients had more effector memory cells and showed sharper drops by 90 minutes. Dynamics were significantly affected by CMV serostatus in CD8^+^ (p=0.05) CD4^+^ T_EM_ (p=0.002), CD4^+^ T_EMRA_ (p=0.002) and CD8^+^ T_EMRA_ (p=0.003). n=52. Horizontal lines display statistical significance across time points for CMV seropositive (red) and seronegative (blue) patients. *=p<0.05; ** = p<0.01; *** = p<0.001; *** = p<0.0001. p values determined using 2-way ANOVA. **(B)** Mean CX_3_CR1 expression on each T-cell subset at 24 hours post-reperfusion.

Coronary MVO is an independent predictor of adverse outcomes after STEMI (16), and we have previously shown an association between MVO and early post-reperfusion loss of effector (CCR7^-^) T-lymphocytes, with cell depletion 15-30 minutes after reperfusion most strongly predictive of MVO (5). We show here that this association is exclusively due to CMV seropositive patients (**Figure 2B**). To account for lower pre-reperfusion counts of effector T-lymphocytes in CMV seronegative patients, we compared the percentage change in cell numbers, rather than absolute, in patients with no, low and high MVO. CD4^+^ T_EMRA_ are effectively absent in CMV seronegative patients (**Figure 3A**) and so we expected to see no changes in this cell population, but they are included in the analysis for completeness. In CMV seropositive patients, the high-MVO group showed a significantly greater percentage drop than the zero-MVO group in all effector memory subsets: CD4^+^ T_EM_ (-23% *vs* -5%; p=0.0001), CD4^+^ T_EMRA_ (-34% *vs* -4%; p=0.0002), CD8^+^ T_EM_ (-26% *vs* – 12.5%; p=0.011) and CD8^+^ T_EMRA_ (-35% *vs* -15%; p=0.021). In CMV seronegative patients, despite a non-significant trend towards greater lymphocyte drop in patients with high MVO than low MVO, there was no significant differences in lymphocyte count changes between the zero-, low- and high-MVO groups despite similar sample sizes in CMV serogroups. CCR7^+^ T-lymphocytes were not associated with MVO in either serogroup.

### CX_3_CR1-Positive T-Lymphocytes Drop Acutely After Reperfusion in CMV Seropositive Patients

Having shown that dynamic changes in effector T-lymphocyte populations after reperfusion predict adverse cMRI findings in CMV seropositive patients, we aimed to characterise these changes in detail (**Figure 3A**). Pre-reperfusion, CMV seropositive patients had significantly higher titres of CCR7^-^ cells - CD4^+^ T_EM_ (cells/µL: 82 *vs* 29, p=0.004), CD4^+^ T_EMRA_ (27 *vs* 4, p=0.001) and CD8^+^ T_EMRA_ (272 *vs* 109, p=0.01). In both serogroups, counts of CCR7^+^ subsets (CD4^+^ T_N_, CD4^+^ T_CM_, CD8^+^ T_N_ and CD8^+^ T_CM_) were all lower prior to reperfusion than at 24 hours, suggesting they have fallen during ischaemia, but did not change in response to reperfusion. In contrast, CD4^+^ T_EM_, CD4^+^ T_EMRA_, CD8^+^ T_EM_ and CD8^+^ T_EMRA_, all of which are CCR7^-^ effector memory cells, behaved differently in CMV seropositive and seronegative patients. In CMV seronegative patients they resembled CCR7^+^ cells, with minimal change in response to reperfusion. In CMV seropositive patients, however, they dropped significantly over the first 90 minutes after reperfusion, returning to near pre-reperfusion levels at 24 hours. At 90 minutes, CMV seropositive patients had lost almost all the excess CD8^+^ T_EM_ that they had prior to reperfusion. This suggests that in CMV seropositive patients, CCR7^-^ effector memory T-lymphocytes do not fall in response to ischaemia (as they were no lower before reperfusion than at 24 hours), but rather they fall in response to reperfusion. Subsets which drop significantly after reperfusion all express CX_3_CR1 (**Figure 3B**), confirming this as a possible mediator of post-reperfusion lymphocyte depletion in CMV-seropositive patients.

### Pre-Reperfusion Lymphopenia in STEMI Patients Correlates With Higher Troponin T Values, and Predicts Long-Term Mortality

**Figure 4:**
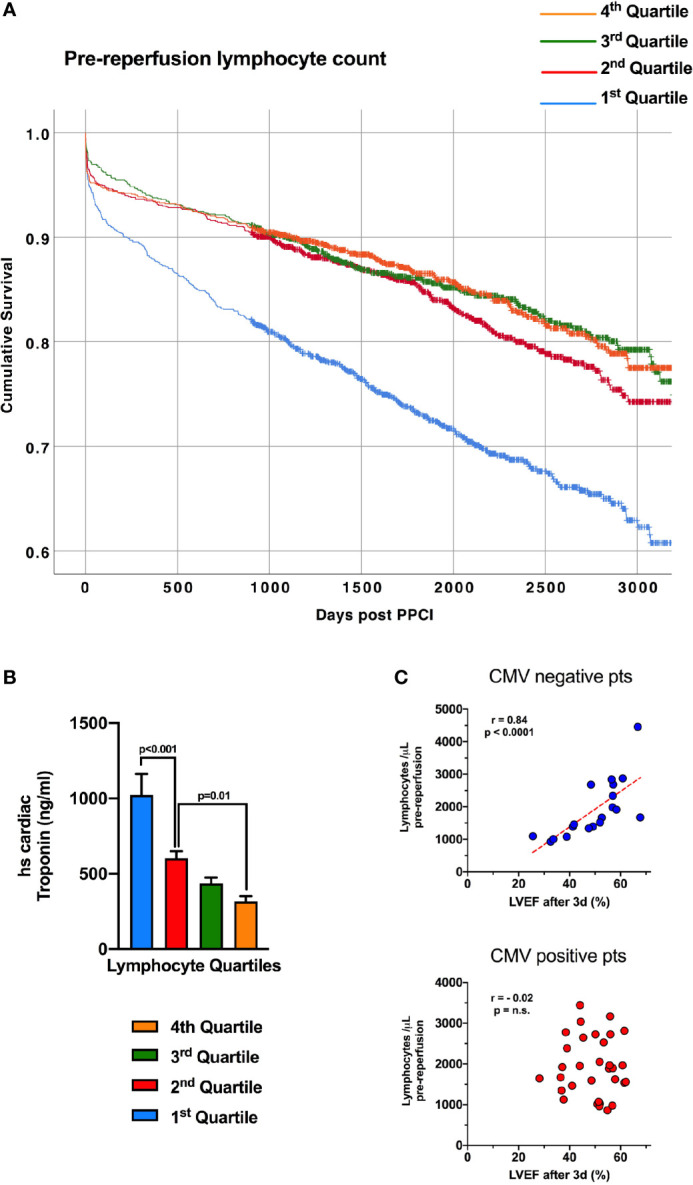
Lymphopenia prior to reperfusion predicts mortality in STEMI and is associated with admission troponin. **(A)** Kaplan-Meier survival curves of 4874 consecutive STEMI/pPCI patients discharged alive following pPCI (mean follow-up time of 7.5 years), divided into four quartiles of pre-reperfusion lymphocyte count, with the lowest quartile having worse survival. Survival of each quartile was compared with unadjusted Kaplan-Meier analysis. **(B)** The same 4874 patients, with the mean admission high-sensitivity cardiac troponin T value (ng/ml) for each lymphocyte quartile, showing that patients with the lowest lymphocytes had the highest troponin values (statistical comparison by unpaired t-tests). **(C)** Correlation between pre-reperfusion lymphocyte count and left-ventricular ejection fraction between 2-7 days post-reperfusion. Lower lymphocyte count was predictive of worse ejection fraction only in CMV seronegative patients.

We have detailed the response of lymphocytes to reperfusion, but assessing dynamic changes due to myocardial infarction itself is more challenging, with pre-ischaemic values rarely available. To investigate whether lymphocytes fall in response to myocardial ischaemia, prior to reperfusion, we analysed the relationship between lymphocyte count and high-sensitivity troponin T (hsTnT) on admission blood samples in 4874 consecutive STEMI patients undergoing pPCI at our center (**Supplemental Table 3**). At admission, prior to pPCI, lower lymphocyte counts correlated with a higher number of anterior infarcts (p<0.001) and higher hsTnT (**Figure 4B**, p<0.001). This suggests that blood lymphocyte counts fall during myocardial infarction, to an extent dependent on the size of the infarct and length of ischaemic time, prior to coronary intervention. Earlier work by our group has shown that lymphopenia one day following reperfusion predicts long-term mortality (5), and here we found that patients with lower lymphocyte counts on admission, prior to reperfusion, also had significantly worse long term survival (hazard ratio for lowest vs highest quartile after adjusting for all covariates: 1.37; 95% CI 1.1-1.7; p=0.004; **Supplemental Table 4** and **Figure 4A**). This divergence in survival curves was evident within a few months and increased throughout the 7.5-year mean follow-up period.

### Pre-Reperfusion Leukocyte Count Correlates With Infarct Size and Left-Ventricular Ejection Fraction in CMV Seronegative Patients

Survival after STEMI is largely determined by left-ventricular ejection fraction (LVEF) (17), so we investigated the relationship between pre-reperfusion lymphocytes and LVEF in 52 STEMI patients (demographics in **Table 1**). Lower pre-reperfusion total lymphocyte count was correlated with lower LVEF at 2-7 days after STEMI, but only in CMV seronegative patients (r=0.839, p<0.001; **Figure 4C**). CMV seropositive patients showed no correlation between admission total lymphocyte count and LVEF (r=0.017). We suggest that this relationship is due to lymphocytes falling in larger infarcts, which lead to worse LVEF – but only in CMV seronegative patients. Interestingly, CCR7^+^ T-lymphocyte subsets all showed significant correlation between pre-reperfusion count and LVEF, again only in CMV seronegative patients (CD4^+^ T_N_: r=0.530; CD4^+^ T_CM_: r=0.502; CD8^+^ T_N_: r=0.689; CD8^+^ T_CM_: r=0.644; data not shown, all p<0.05). NK cells (CD16^+^CD56^+^), CD4^+^ T_EM_ and T_EMRA_ and CD8^+^ T_EM_ and T_EMRA_ did not correlate with LVEF, and none of these cells express CCR7. We have previously looked at 15 different chemokine receptors expressed on lymphocytes (5), and CCR7 did not correlate with post-reperfusion cell drops. Together, this suggests that the mechanism by which pre-reperfusion leucocyte counts fall in STEMI is specific to CCR7^+^ cells, and may not occur in CMV seropositive patients.

### CD3+ T-Lymphocyte Infiltration in Human Failing Hearts

**Figure 5:**
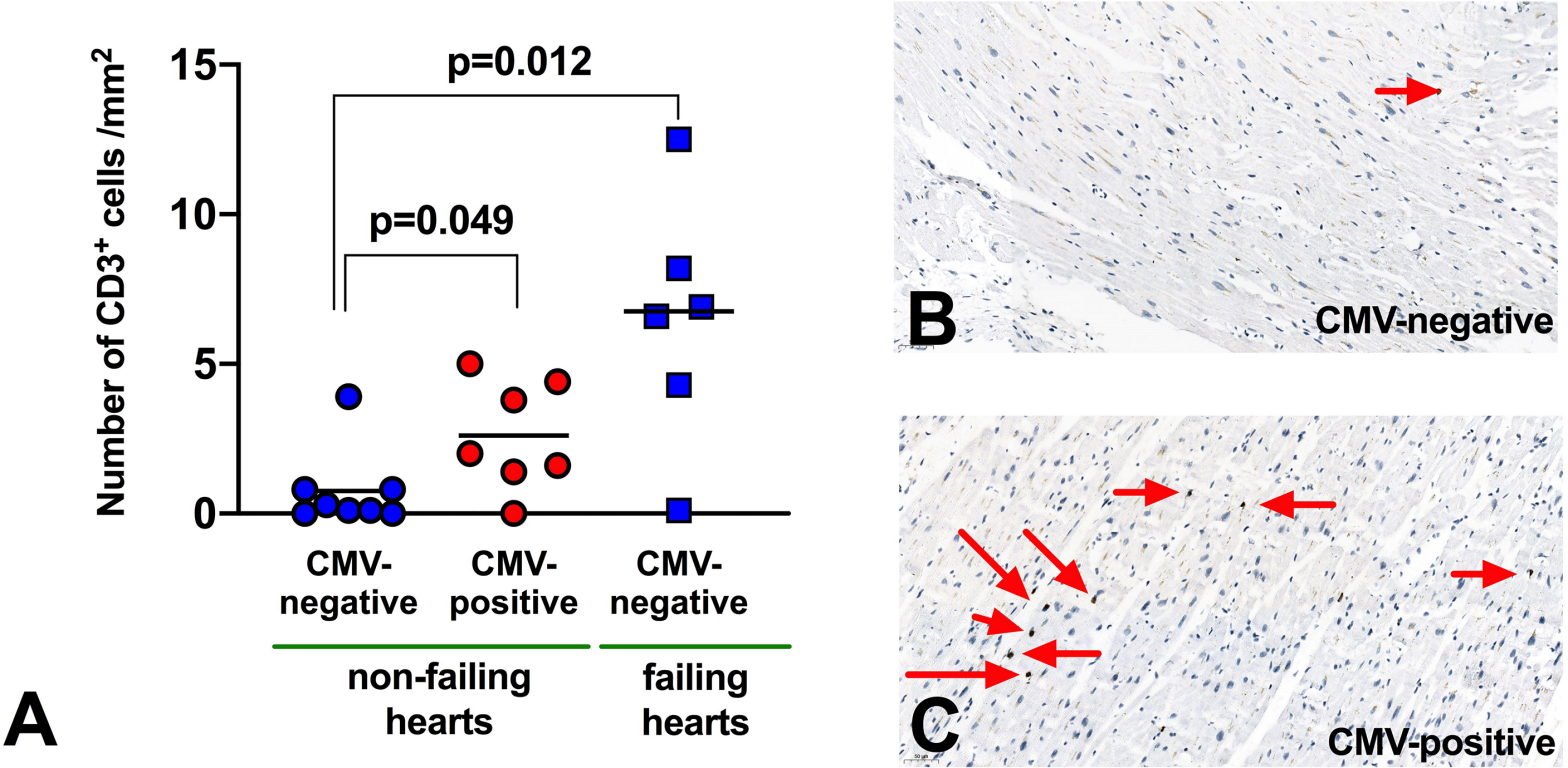
Infiltration of CD3 T-lymphocytes in myocardium. **(A)** Abundance of T-lymphocytes in myocardial tissue from human explanted hearts. n=21 (non-failing hearts: 8 seropositive, 7 seronegative; failing hearts: 6 seronegative). Mann Whitney non-parametric test. **(B, C)** Representative immunohistochemistry to demonstrate CD3^+^ T-lymphocyte infiltration (red arrows) in myocardium from non-failing hearts.

Finally, we analysed 21 explanted hearts (15 non-failing, 6 failing) for infiltration of CD3^+^ T-lymphocytes (**Figure 5**). In non-failing hearts (8 from CMV seropositive donors, 7 from seronegative), we found a significant increase in CD3^+^ T-cells in CMV seropositive hearts (2.6 *vs* 0.75 CD3**^+^** cells/mm^2^, p=0.049), suggesting a role of T-cells for CMV-related myocardial remodeling.

Of interest, in failing hearts (all from seronegative donors) the number of infiltrated T-cells appeared even higher (6.4 CD3**^+^** cells/mm^2^, p=0.012 vs non-failing hearts), but there were too few failing hearts from CMV seropositive donors to compare this meaningfully. We did not have access to paired blood samples to measure peripheral blood T-lymphocytes for these patients.

Another limitation is that, due to the small sample of hearts studied, we were not able to adjust this analysis for demographic differences between CMV serogroups. In particular, CMV seropositive patients are generally older (**Table 1**), and this may be an important confounding variable in our data.

## Discussion

In this study, we describe several observations from varied cohorts. First, we show that remodeling of the left ventricle at 12 weeks after STEMI is worse in CMV seropositive patients.

In a large retrospective cohort, we show that pre-reperfusion lymphopenia is associated with higher troponins and long-term mortality, suggesting that more severe infarcts drive greater lymphopenia even before reperfusion. Detailed analysis of our prospective cohort showed that reduced early LVEF correlated with reduced counts only of CCR7^+^ (non-effector) T-lymphocytes, and only in CMV seronegative patients. We propose that CCR7^+^ T-lymphocytes are depleted prior to reperfusion in CMV seronegative patients, and by a different mechanism CX_3_CR1^+^ T-lymphocytes are depleted after reperfusion in CMV seropositive patients. Finally, in a small sample of explanted non-failing hearts, we found that lymphocytes were more abundant in hearts from CMV seropositive donors – if replicated in a larger cohort, this would support a hypothesis of heightened myocardial inflammation in these patients.

### CX_3_CR1 Is Associated With Post-Reperfusion Lymphocyte Depletion in CMV Seropositive Patients

Having previously shown that post-reperfusion lymphopenia predicts long term mortality in patients following myocardial infarction (5), in this study we show in a very large population of all-comer patients that pre-reperfusion lymphocyte count also predicts mortality, and is inversely correlated with admission troponin. Troponin was used as a universally available surrogate marker of infarct severity, and this association suggests that lymphocytes fall prior to reperfusion, and fall more with larger infarcts and longer ischaemic times (both of which result in high troponin values) (18, 19). In our prospective cohort, pre-reperfusion lymphocyte count also correlated with left-ventricular ejection fraction (LVEF), a marker of prognosis and infarct severity, but only in CMV seronegative patients, and only in CD4^+^ and CD8^+^ T_N_ and T_CM_, which express CCR7. Conversely, CD4^+^ and CD8^+^ T_EM_ and T_EMRA_, which are CCR7-negative, were not correlated with LVEF - strongly suggesting that CCR7^+^ T-lymphocytes are selectively depleted prior to reperfusion. CCR7 is associated with a non-cytotoxic, CX_3_CR1-negative T-cell phenotype and, along with its ligands CCL19 and CCL21, is predominantly involved in trafficking lymphocytes to secondary lymphoid organs (20). Earlier work has shown that CCR7^+^ T-lymphocytes also drop to some degree in the 30 minutes after reperfusion (5, 21), but we do not know whether these are trafficked to lymphoid organs or sequestered within the myocardial vasculature or myocardium itself.

In contrast, T-lymphocyte subsets which are CX_3_CR1^+^ drop in the 90 minutes after reperfusion. The Bolovan-Fritts group has shown that CMV-specific T-lymphocytes strongly induce membrane-bound fractalkine on vascular endothelium (10), which we hypothesise is interacting with CX_3_CR1+ T-lymphocytes and removing them from circulation. We have previously published evidence that T-lymphocytes are lost within the myocardial vasculature during STEMI/pPCI, and our data from human heart explants now suggests that CMV seropositive hearts display greater infiltration of the myocardium by T-lymphocytes, further supporting the theory that lymphocytes are recruited to the myocardium to a greater extent in seropositive patients (5).

### CMV Seropositivity Is Associated With Adverse Left-Ventricular Remodeling After STEMI

The cardiovascular risks conferred by CMV seropositivity remain controversial. Case-control studies, such as that by Siscovick (22), have not identified an association between CMV IgG and the development of cardiovascular disease. As CMV and cardiovascular disease are so prevalent, however, this study design probably lacks power to identify this association, as the authors themselves stated. A 2017 meta-analysis of prospective epidemiological studies estimated a 7-38% increased relative risk of cardiovascular disease in seropositive patients, which the authors calculate would account for 13% of the total cardiovascular disease burden, due to the high prevalence of CMV (23). The impact of CMV on ventricular remodeling after STEMI, however, has not been well studied. We show that CMV seropositive patients display evidence of worse remodeling on cMRI, with ventricular dilatation by 12 weeks after pPCI. This is evidence that latent CMV infection promotes a clinically relevant adverse response to STEMI and pPCI, although the observed effect was small and it is essential that this association is investigated in larger groups of patients, followed over a longer period. A significant association after only 12 weeks could herald a substantial decline in function when measured over years. There is evidence from mouse models of myocardial infarction and reperfusion that a deranged inflammatory response leads to adverse remodeling. The Frangiogiannis group (24) has shown that CCR5 knockout mice are unable to recruit regulatory T-lymphocytes to the infarcted myocardium and display worse remodeling, while Cochain and colleagues showed that the decoy receptor D6, which internalises and defunctions pro-inflammatory chemokines, is necessary for healthy remodeling in the infarcted murine heart (25). We have previously shown that FoxP3**^-^** CD4**^+^** T-lymphocytes significantly contribute to age-related myocardial inflammation in mice (26). In further studies, we found that CMV-seropositive patients demonstrate signs of accelerated immune ageing following myocardial infarction, that seem to link with impaired myocardial healing (27, 28). Accordingly, we suggest that in CMV seropositive human patients, a dysregulated immune response to STEMI and pPCI leads to excessive inflammation, MVO and adverse remodeling.

### Anti-Inflammatory Therapy in Myocardial Infarction as a New Therapeutic Target

The growing literature on the inflammatory component of cardiovascular disease reveals an enormous arsenal of potential therapies. To date, however, treatments targeting the immune component of cardiovascular disease have not been universally successful. The CANTOS trial showed for the first time that specific anti-inflammatory therapy (with canakinumab, an interleukin-1β inhibitor) can improve cardiovascular outcomes in patients with a history of myocardial infarction and evidence of persistent inflammation (1), although the reduction in the primary endpoint of major adverse cardiovascular events was modest, driven by lower risk of non-fatal MI, and the lack of a mortality benefit leaves the CANTOS trial as predominantly a proof-of-concept study. Subsequently, the cardiovascular inflammation reduction trial (CIRT) and the colchicine cardiovascular outcomes study (COLCOT) showed that more readily available, less targeted immunosuppressants (low-dose methotrexate and colchicine, respectively), do not meaningfully improve cardiovascular outcomes (2, 3). Taken together, these studies support further investigation of well-defined immunological pathways as therapeutic targets in cardiovascular disease, but suggest that non-specific anti-inflammatory therapy is unlikely to succeed.

These trials looked at vascular events, but did not address post-MI outcome. Larger clinical trials on anti-inflammatory approaches in post-MI patients are widely lacking. Gu and colleagues have shown that in a murine model of induced myocardial infarction, specific inhibition of fractalkine improves LV function and survival (29). This specific inhibitor has been trialled in patients with rheumatoid arthritis, and was safe and well-tolerated (30). Specific blockade of the fractalkine-CX_3_CR1 axis in humans therefore emerges as a tantalising candidate for a novel adjunctive therapy in STEMI patients treated with pPCI, which may improve left ventricular function and reduce microvascular obstruction, particularly if targeted to CMV seropositive patients.

### Conclusion

Our study provides evidence that the healing process in myocardial infarction is different for patients who are seropositive for previous cytomegalovirus infection. We suggest that additional myocardial inflammation triggered by cytotoxic T-lymphocytes and CX_3_CR1 is more detrimental to recovery, making fractalkine signalling a valid drug target in seropositive patients.

## Data Availability Statement

The raw data supporting the conclusions of this article will be made available by the authors, without undue reservation.

## Ethics Statement

The studies involving human participants were reviewed and approved by National Research Ethics Committee North-East – Newcastle and North Tyneside 2; REC reference: 14/NE/1070 National Research Ethics Service Committee North East; REC reference: 12/NE/0322 National Research Ethics Service Committee East Midlands – Derby; REC reference: 15/EM/0072 National Research Ethics Committee North-East – Newcastle and North Tyneside 1 Research Ethics Committee; REC reference: 18/NE/0178 Ethics committee of the Medical University of Graz (20-277 ex 08/09, 26-282 ex 13/14 and 28-508 ex 15/16). The patients/participants provided their written informed consent to participate in this study.

## Author Contributions 

LS performed analysis of cellular and cMRI data, and wrote the manuscript, with revisions from GR, UH, KSt and IS. cMRI scans were performed and analysed by CP, AM and SB. Cellular data was collected and analysed by CP, SC, PP, SB, KB, KSo and GR. Analysis of explanted human hearts was performed by VS, LR and PR. The study was led by IS. All authors contributed to the article and approved the submitted version.

## Funding

The research was funded/supported by grants from ERA-NET and the Austrian Science Fund (FWF) to P.P.R. (I 4168-B), GR is supported by the Interdisciplinary Center for Clinical Research Würzburg, the ERA-NET-CVD (grant 01KL1902 to GCR), and the German Research Foundation (DFG grant 411619907). IS is funded by the British Heart Foundation (PG/18/25/33587) and National Institute for Health Research (NIHR) Newcastle Biomedical Research Centre based at Newcastle upon Tyne Hospitals NHS Foundation Trust and Newcastle University. The views expressed are those of the author(s) and not necessarily those of the NHS, the NIHR or the Department of Health.

## Conflict of Interest

The authors declare that the research was conducted in the absence of any commercial or financial relationships that could be construed as a potential conflict of interest.
